# Long-term condition management for prisoners: improving the processes between community and prison

**DOI:** 10.1186/s12875-021-01417-9

**Published:** 2021-04-26

**Authors:** NMJ Wright, F Hankins, P Hearty

**Affiliations:** Spectrum Community Health CIC, Wakefield, UK

**Keywords:** Long-term conditions, Prison healthcare, Community healthcare, Quality outcomes framework

## Abstract

**Background:**

Prisoner populations have a disproportionately high prevalence of risk factors for long-term conditions (LTCs), and movement between community and prisons is a period of potential disruption in the ongoing monitoring and management of LTCs.

**Method:**

Nineteen qualitative interviews with staff, recruited by purposive sampling for professional background, were conducted to explore facilitators and barriers to screening, monitoring and medicines management for LTCs.

**Results:**

There is variability in prisoner behaviours regarding bringing community GP-prescribed medication to prison following arrest and detention in police custody, which affects service ability regarding seamless continuation of community prescribing actions. Systems for actively inputting clinical data into existing, nationally agreed, electronic record templates for QOF monitoring are under-developed in prisons and such activity is dependent upon individual “enthusiast(s)”.

**Conclusion:**

There is a pressing need to embed standardised QOF monitoring systems within an integrated community/prison commissioning framework, supported by connectivity between prison and community primary care records, including all activity related to QOF compliance.

## Background

Long-term conditions (LTCs) are those that cannot be cured but are controlled through medication and/or other forms of therapy [1]. It is estimated that more than 15 million people in the UK suffer from a long-term condition [2], with multi-morbidity also becoming increasingly problematic [1].

The risk factors for such LTCs disproportionately affects prisoner populations [3]. Currently in England and Wales there are over 83,000 individuals imprisoned [4]. Compared to equivalent community populations, prisoners consult primary care doctors three times more frequently, consult other primary healthcare workers 80 times more frequently, and receive inpatient care at least 10 times more frequently [5]. They have higher mortality and morbidity rates from chronic disease due to socioeconomic determinants [6, 7].

Internationally, ethical codes of practice highlight a “principle of equivalence” which states that prisoners have a right to an equivalent quality of healthcare as they would receive in the community [8]. However, in practice there are significant threats to providing such equivalent healthcare, for example consultations which may take place without access to GP or hospital records or may be held in an environment that could compromise safety for both patient and doctor [9]. This is particularly commonplace when patients enter remand prisons outside of normal working hours and are assessed in first night prison reception centres. In such an environment there are significant threats to effective medicines management—defined as “a system of processes and behaviours that determines how medicines are used by patients and by the NHS” [10]. Prisoners commonly enter prison without medication prescribed for their LTC and confirming required medication with community GP services is not possible outside of normal working hours. There are few national guidelines to inform best practice in terms of when it is appropriate to offer substitute medications where medication cannot be confirmed on first night reception areas. By implication, there are no guidelines to inform clinicians when it is appropriate to withhold medication pending confirmation by a third party.

Once patients have undergone assessment and are established in prison there is an opportunity to obtain supporting evidence to confirm their self-report of an LTC by obtaining confirmation from either their community GPs or arranging the necessary clinical tests. In community general practice in England, the key framework to achieve this objective is the Quality and Outcomes Framework [11] (in Scotland such information is collected and presented through Primary Care Information Dashboards [12]). Such a national standardised framework is now embedded in community general practice with financial remuneration linked to assessment and ongoing review of key clinical outcomes pertaining to LTCs. However, healthcare in prisons in England is not linked to financial remuneration through the QOF framework and prison clinicians are not mandated to adhere to the framework. Therefore, since compliance with QOF monitoring processes is voluntary in prisons, it is possible that an opportunity to improve clinical outcomes associated with LTCs is being missed.

In response to such a gap in service provision, we explored the topic of the assessment and management of LTCs in four remand prisons. By conducting qualitative interviews we explored facilitators and barriers regarding compliance with both first and second assessment, the QOF monitoring processes and also systems for managing medicines during the time of transition from community to prison.

## Methods

The qualitative phase of this research was part of a larger mixed methods study, in which the findings of the qualitative and quantitative aspects have been reported separately. The quantitative phase related to data extraction and secondary data analysis of routinely collected clinical data pertaining to long-term condition management in a cohort of 1,126 prisoners across four prisons. The quantitative findings have been submitted in the final report for the funding body but are yet to be published.

For the findings reported below, qualitative methodology was employed as the emphasis in qualitative research upon understanding meanings and experiences makes it particularly useful for quality assessment and for unpacking some of the complex issues inherent to quality improvement in healthcare systems [13]. Following a rapid review of the literature pertaining to the management of long-term conditions in prisons and discussion with practising prison based clinicians, NW devised a qualitative interview schedule. The interviews with members of staff explored the following key topic areas: QOF completion in custodial settings; the continuation of community prescribed medication in first night reception centres; time to first medication dispense; time to first medication administration and completion of first and secondary reception screens. After acquiring the necessary national ethics, prison National Research Committee and local governance approvals, 19 qualitative interviews were undertaken by two researchers, FH and PH. Interviews were conducted with staff across four UK remand prisons. Two prisons were female with operational capacity of 371 (Prison 1) and 486 (Prison 4) respectively and two were male with operational capacity of 1060 (Prison 2) and 1212 (Prison 3) respectively. Participants were approached through a combination of face-to-face discussions and email correspondence, and a purposive sample was used to ensure a range of professional backgrounds were represented, including head of healthcare, lead general practitioner, advanced nurse practitioner, LTC nurse, lead pharmacist, pharmacy technician and healthcare assistants. Demographic information for each of the participants can be found in Table 1. None of the staff members approached declined to take part in the interviews and no repeat interviews were conducted. Interviews lasted between 40 min and two hours.

**Table 1:** Demographic details of participants

Participant number	Prison	Role	Length of prison service	Recording technique
Participant 1	2	GP	18 years	Audio-recorded
Participant 2	2	Pharmacy Technician	Just under 3 years	Audio-recorded
Participant 3	2	Long-term conditions nurse	Just under 10 years	Audio-recorded
Participant 4	2	Advanced Nurse Practitioner	1 year	Audio-recorded
Participant 5	4	Matron	10 years	Audio-recorded
Participant 6	4	Agency Nurse	18 years	Audio-recorded
Participant 7	4	Advanced Nurse Practitioner	8 years	Audio-recorded
Participant 8	4	Healthcare Assistant	7 years	Audio-recorded
Participant 9	4	Nurse/Team Leader	9 years	Hand-written notes
Participant 10	3	Pharmacist	Just under 2 years	Audio-recorded
Participant 11	1	Primary Care Lead Nurse	7 and a half years	Audio-recorded
Participant 12	1	Associate Medical Director	5 years	Audio-recorded
Participant 13	3	GP	15 years	Audio-recorded
Participant 14	1	Locum Pharmacist	4 years	Hand-written notes
Participant 15	1	Head of Secure Environments	Just under 2 years	Audio-recorded
Participant 16	3	Primary Care Team Leader	10 and a half years	Audio-recorded
Participant 17	3	Long-term conditions nurse	7 years	Audio-recorded
Participant 18	3	Deputy Head of Healthcare	3 years	Audio-recorded
Participant 19	2	Senior Practice Nurse	9 years	Audio-recorded

There is considerable variability between prisons regarding healthcare staff complements on account of variations in prison size and category status. However, a typical complement for a male remand prison of approximately 1200 prisoners is shown in Table 2. Compared to general practice there tend to be a lower complement of GPs and a higher complement of nursing staff, with a higher relative ratio of advanced nurse practitioner versus GP workforce. Also, regarding substance misuse provision, only healthcare staff are provided in Table 2, i.e. staff providing clinical interventions. Drugs workers who provide psychosocial interventions are not employed by healthcare providers and typically total a further eight staff members.

**Table 2:** Typical staff complement for a 1200 inmate male remand prison (number of whole time equivalents)

Head of healthcare (1)
Performance and Business lead (1) with two support staff—one administrative one a clinical coder
Team Leaders (2)—one clinical and one operational
General Practitioner (1)
Advanced Nurse Practitioner (1)
Senior nurses (4)—two mental health and two physical health
Nurses (12)—six mental health and six physical health
Substance misuse nurses (3)—one senior nurse and team lead with two support nurses
Healthcare support workers (12)
Administrators (7)
Porters (2)

In one of the interview sites, one of the researchers was a former colleague of a number of the staff members interviewed and potential bias was minimised through encouraging the researcher to remain reflexive regarding her research practice in accordance with established qualitative research methodology [14], followed by an independent check of the interview transcripts by the other members of the research team. Participants were provided with participant information sheets and so understood the key aims of the research. Interviews were held in the member of staff’s usual work place, either inside the prison estate, outside the prison in a training building or in a corporate office building.

Participants were asked to sign an Informed Consent Form before the commencement of the interviews. All interviews were semi-structured and conducted in private, using a pilot-tested interview schedule. All but two of the interviews were audio-recorded, the exceptions being not being able to obtain security clearance to use the audio-recorder within the establishment. In these cases, written notes of the interviews were taken by one of the researchers, while the other conducted the interview. Recordings and notes were transcribed and manually (without software) analysed using the thematic approach to analysis, with one of the authors being the key coder and the other as a checker [15]. The researchers ceased the collection of data after they felt that data saturation had been met. The thematic analysis involved a reflexive and recursive process between the following phases to ensure a rigorous process of data interrogation and engagement. First a phase of data familiarisation was undertaken through reading and re-reading the data, to become immersed and intimately familiar with its content. This led to the second phase which was one of coding the data by generating succinct labels to identify important data that could have been relevant to answering the research question. Thirdly, a phase of generating initial themes was undertaken which involved examining the codes to identify significant broader patterns of meaning. Such patterns formed potential themes from which relevant data was collated relevant to each candidate theme. Fourthly a process of reviewing the candidate themes was undertaken, which involved checking the candidate themes against the dataset with an aim to refine the themes such that they accurately reflect the data. Upon review the themes were then named such that they could be written into coherent findings for the readership.

## Results

Key themes that emerged following analysis of the qualitative interviews are presented below.

### Continuation of community prescriptions

The threats to continuing prescriptions initiated in the community upon reception into prison emerged as a key theme and the process of confirming medications with the community GP in an effort to ensure seamless continuity is discussed below.

#### “GP confirmation” of prior community prescribing

“GP confirmation” (GC) is the process whereby a patient’s GP contact details are obtained to enable healthcare staff to obtain current management plans for LTCs. Such ‘confirmation’ is by facsimile transmission and transfers community GP clinical data typically pertaining to prescribing and referral information.

Participants explained that GC is typically received, via fax, in the form of a detailed account from the new prisoner’s previous community doctor confirming any LTCs, medications (both historic and current), and any outstanding appointments with secondary care services. There was variability reported in the quality of information received from community GPs. In the worst scenarios, GC was limited to just a brief overview of the new prisoner’s current conditions and medications.

There was also variability between the prisons in whose role it was to obtain GCs. Participants from Prison 4 reported that pharmacy technicians are tasked with obtaining GCs, whereas participants from Prison 3 reported that administrative healthcare staff are responsible for such tasks. Participants from Prison 2 explained that nurses chase the GC, whereas at Prison 1 it seemed to be the responsibility of healthcare assistants. Crucially, whilst there was no clearly articulated reason for such differences between prisons, there were no apparent differences in the quality of clinical data received according to the professional background of healthcare member communicating with the GP practice.

All participants were aware of at least some of the problems faced by staff when attempting to obtain GC, of which one key theme emerged pertaining to a perception that staff within community general practices tended to be delaying the process. For instance, some community practices do not fax patients’ details back to the prison when requested:

*“…the pharmacist always chased up GP information the next morning and fax all the patient’s…our patient signs their consent to the doctor giving us information and then we get the information from the GP, which is very frustrating because very often we’re waiting a long time for it” (Participant 3)*

It was not clear whether such a delayed response on the part of some community GPs was intentional, but the frustration was reported from participants from each of the four prisons and is articulated by Participant 3:

*“…this patient we’re looking after, on your behalf, we can’t look after them until you let us know, you know it can almost end up getting a bit “snotty” with them, and you don’t want to, but it’s just frustrating. I mean some are very good and it does tend to be the same ones that are very good and the same ones that aren’t, and that makes you realise that they do need a bit of a kick really.”*

#### Processes for seamless continuation of community prescriptions

Participants were asked regarding the process for continuing community prescribed medication for a patient newly received into prison. A narrative emerged pertaining to what circumstances enables such prescribing processes to be easier and more efficient, since within the same prison and on the same evening reception clinic there is variability in individual prisoner behaviours regarding both whether they bring community prescribed medication to prison, and if so in what form of packaging it is presented. The ideal scenario is when a new prisoner brings in a clearly labelled medications blister pack showing the following: prisoner name; medication name; dosage details and packaging highlighting that the medication is not beyond the expiry date. This reportedly makes it easier to manage risk, since decisions can be informed by correct and up-to-date information regarding the new prisoner’s current medication needs. Also, with medication in blister packs it is not possible to substitute with medication that would have currency within a prison since it would entail breaking seals that could not be subsequently resealed. With tablets dispensed into medication bottles, they could be substituted for such medication. Thus, the theme emerged of a readiness to administer medication without delay when it is presented in the correctly labelled blister pack:

*“…we sort of already have the confirmation then, so we can prescribe straightaway” (Participant 7)*

Participants explained that if medication is not received in the form described above then GC is necessary before prescriptions can be issued and dispensed. Therefore, when community General Practices fail to send the GC in a timely manner, prescribing and dispensing can be delayed unnecessarily:

*“…where the practice has been correctly identified but the practice doesn’t play ball, they request consent, the GP consent form and one or two days they haven’t sent what you have requested, that’s an unnecessary delay” (Participant 19)*

Although unnecessary delays to the continuation of medication do occur, some participants emphasised the efforts healthcare staff go to in order to avoid them:

*“…we get the patient’s family or friends, whoever, to actually bring the meds in so we have them” (Participant 10)*

Furthermore, some participants from the two female prisons explained that healthcare staff will even go to the lengths of sourcing medications from external ‘24-h’ pharmacies and sometimes task taxi drivers with the job of collecting and delivering medications. It was not explicitly clear why only two out of the four prisons went to such lengths, but both are female establishments housing a considerably lower number of prisoners. Therefore, it is possible that there was sufficient staff resource to permit this labour-intensive process.

### Medicines management

Issues pertaining to both the process of holding stock medication and accessing out-of-hours medication that is held in prison pharmacies are presented below.

#### Stock medication

Respondents discussed the various medications kept in stock within the four prisons. Across all prisons, it was reported that most stock is made up of the following medications: analgesics; inhalers for the management of asthma; anti-angina sprays; anti-epileptic medications; medication to treat diabetic hypoglycaemic or hyperglycaemic episodes; medications to ameliorate withdrawal symptoms from substance or alcohol use.

Although most participants reported such stock is adequate in meeting acute medical need, it was argued that more medications are needed to ensure continuity of prescribing and that if stocked, waste would be reduced:

*“We have a stock list, it’s very basic…” (Participant 1)*

*“The majority are obviously all your symptomatic relief medications, antibiotics, there are, from what I recall, antidepressants, some but not all. Aspirins, heart medication but it’s quite limited in that sense... Bog standards like your Gaviscon, obviously your methadones, buprenorphine, I think and then obviously your emergency drugs” (Participant 6)*

Whilst some participants articulated that stocklists are based on the prevalence of conditions among the establishment’s prisoners, such a view was not uniform. Rather most participants were unable to explain the process informing which medications are stocked. Such lack of clarity was even illustrated by a pharmacist, whose role it is to manage stock medication. When asked if there is a particular reason behind which medications are stocked, Participant 10 responds:

*“Erm…no, I don’t think there is, I could be wrong, but I don’t think there is anything like that, like I said, if it’s a long-term condition, it tends to be the same drugs that are prescribed all the time, unless they’ve got somebody who doesn’t get on with a particular med and is prescribed something a bit different by a consultant…it’s just the same that you would see if you went into a community pharmacy outside”*

However, Participant 7 – an Advanced Nurse Practitioner from Prison 4 – did seem confident about the reasons that inform medication stock at the first night centre, namely the need to manage acute drug/alcohol withdrawal symptoms:

*“So it’s just, yeah, it’s with a, no, it wouldn’t be disease prevalent so much, but I think, you know, you're primarily tackling, on first night centre, you're tackling life-threatening withdrawal, really. So we’re looking at substance misuse rather than long-term conditions.”*

Although most participants reported such stock is adequate in meeting acute medical need, it was argued that more medications are needed to ensure continuity of prescribing and that if stocked, both medication waste, and time spent accessing prison pharmacies out of hours would be reduced:

*Researcher: If there was something that you knew was in pharmacy, but it wasn’t on the first night, could you get that?*

*Participant: We can get it. Security is quite tight, the doctor or the nurse in charge would have to write a memo to the gate and request the governor’s permission to get the keys to go into pharmacy and it’s not good practice for one individual to go into pharmacy for fairly obvious reasons, so it takes 2 members of staff out and it’s quite a lot of hassle and we’re already running on fairly minimal staffing so we would only do that if there is a real clinical need. For instance, I’ve done it recently for an HIV positive patient because we don’t want to have a gap in their medication because that can cause viral resistance, so I made the decision that it was necessary to go into the pharmacy, that was at Leeds.*

*Researcher: When you say for obvious reasons, just to clarify for the tape, is it because they can take…*

*Participant: They might be accused of taking medication with street value.” (Participant 13)*

### QOF compliance in prison

Issues pertaining to compliance with QOF monitoring in prison settings are presented below.

#### QOF staff knowledge / understanding

When participants were asked about QOF completion in prisons, there was no system across healthcare to facilitate such monitoring. Rather, completion tended to be driven by a small number of participants with senior prescribing skills (i.e. GPs and Advanced Nurse Practitioners) who had substantial knowledge regarding the purpose and utility of QOF monitoring:

*“…when I was the GP, probably more when I was the lead GP here, I drove the QOF agenda, I spent hours of my own time going through people’s records to see if I could repopulate QOF unmet needs to get the QOF points up and at one point we had almost all the QOF points that we reasonably could…” (Participant 13)*

By contrast, healthcare staff who did not have a prescribing remit were either unaware of the process of QOF monitoring or felt that it was not a prison healthcare responsibility to undertake. Such a lack of role legitimacy was highlighted by the following view provided by a senior practice nurse:

*Researcher: “Do you know anything about QoFs?”*

*Participant 19: “I have a rough idea about QoFs but don’t ask me that now because we are still at a very, very infantry stage on QoFs in this establishment”*

*Researcher: “So it is not actually being used right now”?*

*Participant 19: “We are aware of them, they get mentioned, but we are not yet at the stage where I can say or have a discussion on it, no”.*

#### QOF completion barriers

In addition to the view expressed above that there was little point completing QOF templates as there was no compulsion to meet QOF targets, staff commented that they didn’t have time to undertake QOF clinical activity. Such a barrier tended to be highlighted by participants from a nursing professional background:

*“Well, I think it should really be, you know, within the week of them telling us. Ideally. But I think, in reality, you know, our use of QOF tools is probably not as good as it could be. And I think that’s, you know, resource, people are out of practice, staff turnover… Training issues and it’s time constraints as well” (Participant 7)*

High prisoner turnover was also identified as a barrier to undertaking QOF activity:

*“There’s often been talk of QOF in prions but QOF is based on a yearly assessment of your chronic disease, and most of our prisoners aren’t here for a year so your stats would permanently be changed. You know QOF is based on what percentage of your diabetics have had their relevant checks done, but if the vast majority of those don’t stay with you over a year, then you can’t improve your stats, your stats are at the mercy of the fact the prisoners move on" (Participant 1)*

The issue of high prisoner turnover being more of a barrier in male remand prisons was highlighted by Participant 1:

*“I can’t comment on a different gender prison, because I’ve never worked in a female prison, but turnover is a major problem, I mean we get a turnover of 50 prisoners a day so we have 1037 prisons here now, but in a year, we’ll have 4000 people through our doors, so it’s a huge turnover; in a previous study that I saw from our prison, the average stay of prisoners is about 80 days, so you can do a lot in those 80 days but you can’t really improve massively chronic disease, you can stabilise people and you can educate them and put the wheels in motion but you can’t monitor it long-term…as far as long-term prevalence goes we don’t have the stats for that.”*

GP participants tended to be more supportive regarding the use of QOF monitoring in prisons:

*“…it [QOF] did set a standard, an agreed standard for ensuring that the right questions were asked in a consistent way, and the right tasks were done in a consistent way and it cross-referenced that with community general practice as well because we were working to the same sort of QOF standards, so I think its main advantage was that (inaudible) to a nationally agreed consistency for approach to conditions” (Participant 12)*

Many participants talked about alternative methods of recording information pertaining to prisoners’ LTCs. It was revealed that some prison healthcare departments preferred to create and complete their own LTC templates in place of QOF templates:

*“…they’re all getting done, but they’re getting done under the templates as opposed to the QOFs…The senior matron did them for the whole directorate. He made the templates” (Participant 4)*

#### Benefits of access to community QOF and wider clinical data

The majority of participants felt that having sight of community QOF data (through linked community and prison GP records) would be invaluable and offer a range of benefits to healthcare staff within prisons. Some participants explained how such visibility could help to reduce duplication of QOF-related work:

*“I would prefer us to be managing those around need rather than just well our process is that everybody comes in who has diabetes or asthma or whatever goes into this clinic within, again within that period of time, if we could see that somebody, you know three months ago had had their chronic disease management reviewed and the tests done and everything was, was ok, then I don’t see that there’s any reason to then do that until it is due to follow on from that" (Participant 12)*

Participants also identified potential benefits to patients in prison, citing that less hospital appointments would be missed and LTC reviews would be conducted on time to better monitor prisoners’ health. For instance, by being able to see community QOF data, prison clinicians would have access to information regarding patients’ last reviews for any conditions they may have. Therefore, they would see when their next review is due and schedule a prison clinician to conduct this.

*“…it’s quite good for things like registers and people needing flu vacs and the percentages that have had it and things like that” (Participant 15)*

## Discussion

### Summary

Our findings highlight significant variability between community GP practices regarding timeliness of response to requests from prison healthcare departments for clinical information. We also found differences regarding the professional role of the staff member retrieving GC, although the quality of information received did not appear to differ according to the professional background of the staff member.

Our findings highlighted variability in prisoner behaviours regarding bringing community prescribed medication to prison. Where the patient was received into prison accompanied by their community prescribed medication in named patient blister packs it facilitated seamless continuation of community prescribing actions.

It was also highlighted that none of the four prisons had systems for actively inputting clinical data into existing templates for QOF monitoring, and therefore completion tended to be dependent upon an individual “enthusiast(s)”, and management of LTCs was not seen as everyone’s business. High prisoner turnover, time pressures and lack of role legitimacy were highlighted as significant barriers to QOF monitoring. Some prisons developed their own local systems for monitoring of LTCs (in some circumstances this was in addition to using national QOF monitoring templates).

### Strengths and limitations

As far as we are aware, this research is the first study exploring, through qualitative methodology, existing processes for the management of LTCs at point of transition from community to prison general practice, and the potential for QOF to enhance the management of LTCs in UK prisons. Whilst our study took place in four remand prisons, we are confident that our findings can be generalised across the remand prison estate and training prisons, since all prisoners in such establishments have at some point been transferred from remand prisons. However, we would be cautious of extrapolating our findings outside of the UK setting since QOF frameworks are not implemented internationally.

### Comparison with existing literature

Our research highlighted poor compliance with nationally validated QOF monitoring. By contrast, whilst participation in the community QOF is voluntary, participation rates are very high at 94.8% [16]. Also highlighted, was limited medication stocks on prison wings, necessitating a need to occasionally access prison pharmacy services out of hours in circumstances where the patient’s medication is known but not available. Such a process is time consuming and fraught with risks inherent in accessing a site where medications (including controlled drugs) are housed. Therefore, there is merit in implementing and evaluating “proof of concept” pilots of automated systems for medication storage and administration. Over the last 10–15 years automated medication storage systems have been used in the health service, that can lead to a reduction in errors, increased space efficiency and a dramatic increase in time efficiency [17]. However, uptake has been slow and in 2007, the then Healthcare Commission expressed concern at the slow rate of progress in embracing and introducing such new technology and suggested that it should be addressed at the earliest opportunity [18]. Such a statement remains relevant since, at the time of writing, robotic technology is in development and in the near future such technology is likely to play an increasing role in medication dispensing across health services [19].

### Implications for research and/or practice

Since quality of information received by GC did not appear to differ according to the professional background of the staff member, we would suggest that significant human resource savings could be made if this function were delegated to administrative staff universally across the national prison estate. As with any new role, there could be a need for training in this regard of administrative staff groups. We recommend Prisoner Patient Awareness initiatives should be developed and implemented. Such initiatives should have the expressed objective of encouraging patients not to remove labelling from blister packaging (since this is a common behaviour of patients in the criminal justice system where medication is often freely traded in shadow economies) and to either retrieve their medication at point of arrest, or to ensure that a close family member or friend brings their medication as soon as possible following detention. Our research also highlighted an opportunity to develop national prison formularies and stock medication lists to ensure consistency of stock across the prison estate.

Our research highlighted a lack of engagement in QOF monitoring despite a national requirement of prison providers through the Health and Justice Indicators of Performance (HJIPs) to report on QOF data [20]. Therefore, there appears to be a disconnect between national reporting requirements and clinical activity “on the ground”. Arguably, this is due to the fact that financial penalties do not flow for non-compliance with QOF reporting and also that such reporting relies solely upon self-assessment of LTC monitoring. Such issues could be addressed, in part, through the pending developments in the electronic patient record linkage systems, whereby community and prison GP electronic clinical records will be better linked thus highlighting QOF completion due dates to prison clinicians from entries made in the community. Such a prospect was universally welcomed by participants and it was felt that such a development would support seamless monitoring of QOF activity between community and prison. This presents an opportunity to introduce QOF monitoring systems, supported by an integrated community/prison commissioning framework, supported by clinical advice from those experienced in both prison and community provision to support prison clinicians in what would be a new role of undertaking QOF monitoring activities.

Finally, our research highlighted a significant barrier to the seamless continuity of community prescribing when patients enter prison since the current system of electronic record transfer between community general practices [21] does not extend to prisons. It is possible this could be addressed in part through the pending NHS roll-out of new functionalities to its main medical records database through the TPP (‘SystmOne’) provider of electronic clinical records. One of the new functionalities will be the ability to transfer a new prisoner’s GP record from their community GP practice into the prison they are being detained in. Therefore, subject to appropriate processes of informed consent by the patient, the task of requesting and waiting for GP records could instantly become an obsolete practice or be limited to just transfer of large documents. The newly received prisoner’s full GP record will be visible to clinicians in the prison so that they can make safer decisions regarding their care from a more informed perspective, improving continuity and equivalence of care. Such an integrated prison and community record would also facilitate the formal implementation of QOF monitoring into prison healthcare services with the possibility of “payment by results” frameworks that are equivalent to those currently in operation in community general practice.

## Conclusions

There are significant barriers in facilitating seamless management of LTCs between community and prison settings. Recent developments in electronic patient record transfer and electronic medicines management systems presents a window of opportunity to improve the processes for assessment and management of long-term conditions at this interface.

## Data Availability

The datasets used and/or analysed during the current study are available from the corresponding author on reasonable request.

## Abbreviations

LTC: Long Term Conditions
NHS: National Health Service
GP: General Practitioner
QOF: Quality Outcomes Framework
GC: GP Confirmation
